# Nomenclatural changes in the tribe Empoascini of the subfamily Typhlocybinae (Hemiptera, Cicadellidae)

**DOI:** 10.3897/zookeys.346.6392

**Published:** 2013-11-01

**Authors:** Dao-zheng Qin, Si-han Lu, Li-fang Zheng, Yi-xin Huang

**Affiliations:** 1 Key Laboratory of Plant Protection Resources and Pest Management of Ministry of Education, Entomological Museum, Northwest A & F University, Yangling, Shaanxi Province, 712100, China

**Keywords:** Homoptera, Auchenorryncha, taxonomy, leafhopper, synonym, China

## Abstract

One genus and species are synonymized in the tribe Empoascini of the subfamily Typhlocybinae. *Bhatasca* Dworakowska, 1995is a junior synonym of *Alebrasca* Hayashi & Okada, 1994, *Bhatasca rectangulata* Qin & Zhang, 2011is a junior synonym of *Alebrasca actinidiae* Hayashi & Okada, 1994. Furthermore, *Bhatasca expansa* is (necessarily) transferred to the genus *Alebrasca*.

## Introduction

Empoascini is a large tribe in the leafhopper subfamily Typhlocybinae, comprising more than 1000 species and nearly 70 genera worldwide. However, the status of some established genera and species in this tribe remains dubious or misleading and needs further revision. In this study, one genus and one species are recognized as junior synonyms and we also propose one new combination in Empoascini. The purpose of the present paper is to clarify the taxonomy of the tribe.

## Nomenclatural changes and notes

### Family Cicadellidae
Subfamily Typhlocybinae
Tribe Empoascini

#### Alebrasca

Hayashi & Okada, 1994

http://species-id.net/wiki/Alebrasca

Alebrasca Hayashi & Okada, 1994: 267. Type species: *Alebrasca actinidiae* Hayashi & Okada, 1994, by original designation.
Bhatasca Dworakowska, 1995: 143. Type species: *Bhatasca expansa* Dworakowska, 1995, by original designation. **syn. n.**

##### Remarks.

Dworakowska (1995) established the genus *Bhatasca* based on the type species *Bhatasca expansa* Dworakowska, 1995 from Tsyr-feng in Taiwan, and Qin et al. (2011) described *Bhatasca rectangulata* based on the generic diagnosis provided by Dworakowska (1995). Until now, only two species were included in the genus, widely distributed in mainland China (Hunan, Henan, Gansu, Sichuan, Fujian, Zhejiang, Jiangxi) and Taiwan. Based on investigation of the descriptions and illustrations by Hayashi and Okada (1994) and Dworakowska (1995) and *Bhatasca* material deposited in the Entomological Museum, Northwest A&F University (NWAFU), we recognize the genus *Bhatasca* Dworakowska is a junior synonym of *Alebrasca* Hayashi & Okada.

#### Alebrasca actinidiae

Hayashi & Okada, 1994

http://species-id.net/wiki/Alebrasca_actinidiae

Alebrasca actinidiae Hayashi & Okada, 1994: 269.
Bhatasca rectangulata Qin & Zhang, 2011: 54. **syn. n.**

##### Remarks.

After checking the type specimens of *Bhatasca rectangulata*, we found the characters of wing, basal abdominal apodemes and male genitalia described by Qin et al. (2011) in mainland China are the same as those of the species described by Hayashi and Okada (1994). Furthermore, the distribution of *Bhatasca rectangulata* has nearly same latitude as the type locality of *Alebrasca actinidiae* Hayashi & Okada in Japan (central Honshu). Therefore, synonymy of the two species is confirmed here.

#### Alebrasca expansa

(Dworakowska, 1995) comb. n.

http://species-id.net/wiki/Alebrasca_expansa

Bhatasca expansa Dworakowska, 1995: 145.

##### Remarks.

Dworakowska (1995) described this species in *Bhatasca* from Taiwan. We here transfer it to *Alebrasca* mainlybased on the characters of the head, the venation of fore- and hindwing, the basal abdominal apodemes and especially the configuration of the male genitalia.

## Supplementary Material

XML Treatment for Alebrasca

XML Treatment for Alebrasca actinidiae

XML Treatment for Alebrasca expansa
